# Diversified gut microbiota in newborns of mothers with gestational diabetes mellitus

**DOI:** 10.1371/journal.pone.0205695

**Published:** 2018-10-17

**Authors:** Minglian Su, Yuanyang Nie, Ruocheng Shao, Shihao Duan, Youhui Jiang, Mingyue Wang, Zhichao Xing, Qun Sun, Xinghui Liu, Wenming Xu

**Affiliations:** 1 Joint Laboratory of Reproductive Medicine, SCU-CUHK, Key Laboratory of Obstetric, Gynecologic and Pediatric Diseases and Birth Defects of Ministry of Education, West China Second University Hospital, Sichuan University, Chengdu, P. R. China; 2 Department of Obstetric and Gynecologic Diseases, West China Second University Hospital, Sichuan University, Chengdu, P. R. China; 3 West China School of Clinical Medicine, Sichuan University, Chengdu, P. R. China; 4 Postdoctoral Research Base, Henan Institute of Science and Technology, Xinxiang, P. R. China; 5 College of Life Sciences, Sichuan University, Chengdu, P. R. China; Texas A&M University, UNITED STATES

## Abstract

Gestational diabetes mellitus (GDM), a high-risk pregnancy complication of great effect on the perinatal health of women and newborns, may cause changes of gut microbiota in mothers and further affect gut microbiota in newborns. This study aimed to investigate the potential effect of mother GDM on newborns’ gut microbiota. Meconium DNA was extracted from a total of 34 full-term and C-sectioned newborns, in which 20 newborns had mothers diagnosed with GDM, while 14 had unaffected mothers. Sequencing and bioinformatics analysis of 16S rRNA indicated that the gut microbiota of GDM newborns showed differences compared to control newborns. The taxonomy analyses suggested that the overall bacterial content significantly differed by maternal diabetes status, with the microbiome of the GDM group showing lower alpha-diversity than that of control group. The phyla of Proteobacteria and Actinobacteria in GDM newborns increased, while that of Bacteroidetes significantly reduced (*P*<0.05). Moreover, several unique gut microbiota in phylum of Proteobacteria, Firmicutes, Actinobacteria, Bacteroidetes, Chloroflexi, Acidobacteria, and Planctomycetes found in control newborns were absent in GDM ones. At genus level, the relative abundance of *Prevotella* and *Lactobacillus* significantly decreased (*P*<0.05) in GDM newborns. Correlation analysis indicated that maternal fasting glucose levels were positively correlated with the relative abundance of phylum Actinobacteria and genus *Acinetobacter*, while negatively correlated with that of phylum Bacteroidetes and genus *Prevotella*. However, bacteria in GDM grade A2 (GDM_A2) newborns did not show any statistical variation compared to those from control newborns, which might be attributed to the additional intervention by insulin. The results of this study have important implications for understanding the potential effects of GDM on the gut microbiota of newborns and thus possibly their metabolism at later stages in their lives.

## Introduction

Gestational diabetes mellitus (GDM) is an asymptomatic disorder characterized by carbohydrate intolerance, which appears to be normal for glucose metabolism before pregnancy, but leading to diabetes during pregnancy [1]. Because of its high incidence and great influence on mothers and infants, GDM has attracted wide attention worldwide. With the increase of obesity and type 2 diabetes, GDM has increased significantly in recent years. In accordance with epidemiological surveys conducted in China, the incidence of GDM has increased by nearly 3 times, from 2.4% in 1999 to 6.8% in 2008 [2], further reaching 9.3% in 2012 [3]. GDM-affected pregnancies are considered as high-risk, as they are associated with increased risk of preeclampsia, preterm birth and other adverse short term pregnancy outcomes [4]. Long term complications for GDM mothers include increased risk of type 2 diabetes (T2D) and cardiovascular diseases [5, 6]. Additionally, GDM not only increases the risk of adverse pregnancy outcomes in mothers, but also increases the risk of short- and long-term complications in newborns, such as neonatal malformations [7] and neonatal obesity [8]. Moreover, GDM induces long-term neurological defects in infants, which become more pronounced in childhood [9], characterized by attention deficit, lower level of cognition, compromised movement ability or linguistic competence [10].

The foetal gut was thought to be sterile, but existing studies show that bacteria are present in the gut before birth [11]. Early life is the critical period for colonization and maturation of gut microbiota, which can influence the maturation of the newborn's immune system [12, 13], and subsequent development of diseases, such as asthma [14] and obesity [15–17]. The intestinal microbiota in early life is susceptible to the influence of maternal factors, delivery mode [18] and postnatal factors. Studies have shown that the bacterial composition of the maternal gut could affect (both qualitatively and quantitatively) the bacterial content of infant meconium [19], and another investigation also indicated that the placental microbiome profiles were most akin to the nonpregnant human oral microbiome [20]. A previous study has also confirmed that GDM is capable of causing gut microbiota dysbiosis and functional changes in the mother's intestine [21], compared with healthy pregnant women. Accordingly, we hypothesized that gut microbiota from the offspring of mothers with GDM may change and may have impact on the health of offspring. The aim of this study was to investigate the potential correlation between maternal GDM and foetal gut microbiota by exploring the distribution of the dominant microbes in infants.

## Materials and methods

### Ethics

The present study conforms to the principles outlined in the Declaration of Helsinki. This study was approved by the West China Second Hospital Review Board of Sichuan University, Chengdu, China. All patients provided written informed consent prior to participation in the study protocol.

### Study population and sample collection

This study was performed on normal full-term newborns born with caesarean section in West China Second Hospital of Sichuan University from August 31, 2016 to January 10, 2017, and whose mothers provided informed consents. The inclusion criteria were: 1) pregnant women whose gestational age was 37 to 42 weeks and eventually underwent caesarean section; 2) pregnant women who had regular prenatal visits and all clinical information could be obtained; 3) pregnant women with GDM or without GDM. 4) The newborns were full-term. The exclusion criteria were: 1) any infection occurring during pregnancy, especially gastrointestinal or genital tract infections; 2) pregnant women using any type of antibiotic during pregnancy, excluding the antibiotic administered before caesarean section as standard obstetric care; 3) pregnant women with any other complications, such as mental disorder, hypertension, preeclampsia, eclampsia; 4) newborns administrated any type of antibiotic after birth; 5) women who eventually underwent C-section and received an immediate dose of Kefzol (cefazolin) 30 mins prior to C-section as a standard of care were retained in the study; 6) obstetric risks, such as HIV positive, significant congenital anomalies, neurological dysfunction, foetal chromosomal anomalies, or inborn metabolic disorders. The following maternal clinical information was collected: mother's age at childbirth; oral glucose tolerance test in the second trimester (fasting, one hour postprandial, two hours postprandial); whether there was a diagnosis of GDM; whether diabetes was diagnosed before pregnancy; treatment measures for diabetic gravidae; pre-delivery weight; pre-pregnancy and prenatal BMI; gestational weight gain; pregnancy history; gestational age at delivery; delivery mode. Collected infants’ clinical information included sex, weight and height. The personal information was recorded on the infusion label, including the name of the expectant mother, the date of delivery, the registration number, the bed number. The label was attached to the medical waste bags that were distributed to the participants. The samples were taken by collecting the newborn’s diapers with faeces using bags within 24 hours after birth. The meconium was scraped with a disposable blade, collected into 1.5 mL sterilized centrifuge tubes in the examination room and stored at -80°C immediately for later use.

### Faecal DNA extraction

QIAamp DNA Stool Mini Kits (QIAGEN) were used to extract the total DNA of microorganisms in 200 mg of each faecal sample, according to the suggested specific operating procedures recommended by the QIAamp DNA Stool Handbook. DNA concentration was determined with a NanoDrop ND-1000 spectrophotometer (NanoDrop Technologies). The extracted DNA was stored at −20°C until further analysis.

### Microbiome profiling

Meconium microbiome profiles were assessed by 16S rRNA gene amplicon sequencing using the Illumina MiSeq system (Illumina, San Diego, CA, USA). The V4 variable regions of bacterial 16S rRNA genes were PCR amplified from genomic DNA using the primers 515F (5’-GTG CCA GCM GCC GCG GTA A -3’) and 909R (5’-CCC CGY CAA TTC MTT TRA GT -3’) [22]. The PCR mixture (25 μL) contained 1× PCR buffer, 1.5 mM MgCl_2_, each deoxynucleotide triphosphate at 0.4 μM, each primer at 1.0 μM and 0.5 U of Ex Taq (TaKaRa, Dalian) and 10 ng faecal genomic DNA. The PCR amplification program included initial denaturation at 94°C for 3 min, followed by 30 cycles at 94°C for 40 s, 56°C for 60 s, and 72°C for 60 s, and a final extension at 72°C for 10 min. We conducted two PCR reactions for each sample, and combined them together after PCR amplification. PCR products were detected by 1% of agarose gel electrophoresis, purified using the Gel Extraction Kit (Sangon Biotech, Shanghai), determined with a NanoDrop ND-1000 spectrophotometer (NanoDrop Technologies), and finally submitted to Biobit Biotech Inc. (Chengdu, China) for Illumina paired-end library preparation, cluster generation, and 250-bp paired-end sequencing on an Illumina MiSeq instrument in two separate runs.

Raw Illumina fastq files were demultiplexed, in which the paired-end sequences were merged as raw tags using FLASH (Fast Length Adjustment of Short reads) v1.2.7. Then, these raw tags were strictly quality filtered and analysed using QIIME (Quantitative Insights into Microbial Ecology) v1.9.0. Reads were truncated at any site containing more than three consecutive bases receiving a quality score <1e-5, and any read containing one or more ambiguous base calls was discarded. Operational taxonomic units (OTUs) were assigned using QIIME’s uclust-based open-reference OTU-picking workflow, with a threshold of 97% pairwise identity. Sequences were mapped, yielding an OTU table, and a phylogenetic tree was constructed using FastTree [23], which was then used to generate unweighted and weighted UniFrac distance metrics. Any OTUs with overall relative abundance <1e-4 were excluded from further analysis.

### Statistical analysis

Comparisons between the control and GDM group were performed using student *t* test and Mann-Whitney test. Statistical analysis of the clinical data was performed using SPSS (Statistic Package for Social Science) 17.0 software (SPSS Inc., Chicago, IL, USA). *P*<0.05 was considered statistically significant. The gut microbiota diversity within samples from each individual (α-diversity) was assessed by the Shannon index, Simpson index, Chao1 richness index and Observed Species. Significant differences in microbial community composition between the control and GDM group (β-diversity) were calculated by permutational MANOVA (pMANOVA) of weighted UniFrac distances (Bray-Curtis distance method) [24, 25] with 999 permutations and illustrated by principal coordinates analysis (PCoA). Associations between maternal clinical index and gut microbiota in meconium samples of newborns were evaluated by bootstrapped Spearman rank correlation coefficient using 1,000 permutations.

## Results

### Clinical information

In this study, the diagnostic criteria for GDM were based on the GDM diagnostic recommendations of the International Association of Diabetes and Pregnancy Study Groups (IADPSG) [26], and included the 75 g Oral Glucose Tolerance Test (OGTT), fasting, 1 hour and 2 hours post prandial blood glucose values of 5.1 mmol/L, 10 mmol/L, and 8.5 mmol/L, respectively. Any of the above-mentioned blood glucose levels that meet or exceed the diagnostic criteria leads to the diagnosis of GDM. The GDM staging criteria were based on the White classification method [27]. The clinical characteristics of pregnant women and their newborns included in this study are presented in Table 1. A total of 34 newborns were included in the trial, with 20 newborns whose mothers were diagnosed with GDM. Fifteen mothers were diagnosed as GDM grade A1 (GDM_A1) and 5 mothers were diagnosed as GDM grade A2 (GDM_A2). Exercise and diet control were used to treat pregnant women with GDM. Pregnant women with GDM grade A2 were additionally treated with insulin administration. Fourteen newborns from non-GDM mothers were used as the control group. All the newborns included were born by caesarean section, and their neonatal Apgar scores for 1 min, 5 min and 10 min were all 10.

**Table 1:** Clinical information of mothers and newborns.

Items	Control (n=14)	GDM_A1 (n=15)	*P* value	GDM_A2 (n=5)	*P* value
**Maternal characteristics**					
Age (years)	33.64±5.69	36.20±4.49	0.189	35.60±3.65	0.487
Gestational age at delivery (week)	38.93±0.62	38.53±1.06	0.229	39.00±0.00	0.802
Antepartum weight (kg)	67.85±3.80	71.19±6.74	0.126	71.80±7.36	0.148
Gestational weight gain (kg)	12.58±3.86	13.53±6.06	0.629	11.40±5.37	0.609
Pre-pregnancy BMI	21.67±2.12	22.41±2.33	0.386	23.05±3.18	0.295
Antepartum BMI	26.59±1.66	27.71±2.90	0.226	27.33±1.91	0.424
OGTT on an empty stomach (mmol/L)	4.43±0.34	4.85±0.37	0.006	4.85±0.42	0.046
OGTT at postprandial one hour (mmol/L)	7.90±0.94	9.69±1.40	0.001	10.88±0.43	<0.001
OGTT at postprandial two hour (mmol/L)	6.90±0.95	8.97±1.16	<0.001	9.46±1.85	0.002
**Newborn characteristics**					
Weight at birth (kg)	3.39±0.36	3.24±0.55	0.387	3.35±0.31	0.796
Height at birth (cm)	49.43±1.56	49.40±2.59	0.972	49.20±0.84	0.761
Sex ratio at birth (male/female)	1.80	2.75		1.50	

GDM_A1, GDM grade A1; GDM_A2, GDM grade A2; BMI, body mass index; OGTT, Oral Glucose Tolerance Test. Data are expressed as the mean ± SD. The statistically significant difference between the control group and GDM_A1 group or GDM_A2 group are *P*<0.05.

There were significant differences (*P*<0.05) in fasting, 1 hour and 2 hours of blood glucose values between the two groups (Table 1), with GDM mothers having a higher OGTT index. No significant difference was observed in age, gestational age at delivery, antepartum weight, gestational weight gain, pre-pregnancy BMI and antepartum BMI of mothers between control, GDM_A1 and GDM_A2 groups (*P*>0.05, t-test). The newborn weight and height at birth did not differ significantly among the three groups (*P*>0.05, t-test). There was a higher percentage of male newborns in the GDM_A1 group (male/female = 2.75).

### Overview of gut bacterial diversities in the GDM newborns and the control newborn subjects

Rarefaction curve can be used to compare the abundance of species in samples with different numbers of sequences, and to indicate whether the sampling size (sequencing depth) is reasonable. When the curve tends to be flat, it indicates that the number of samples is reasonable, and more samples will produce only a small amount of new OTU. Conversely, more new OTU may be generated by continuing the sampling. According to the Figure A in S1 File, 10,357 sequences per sample of sequencing depth was reasonable.

**Fig 1:**
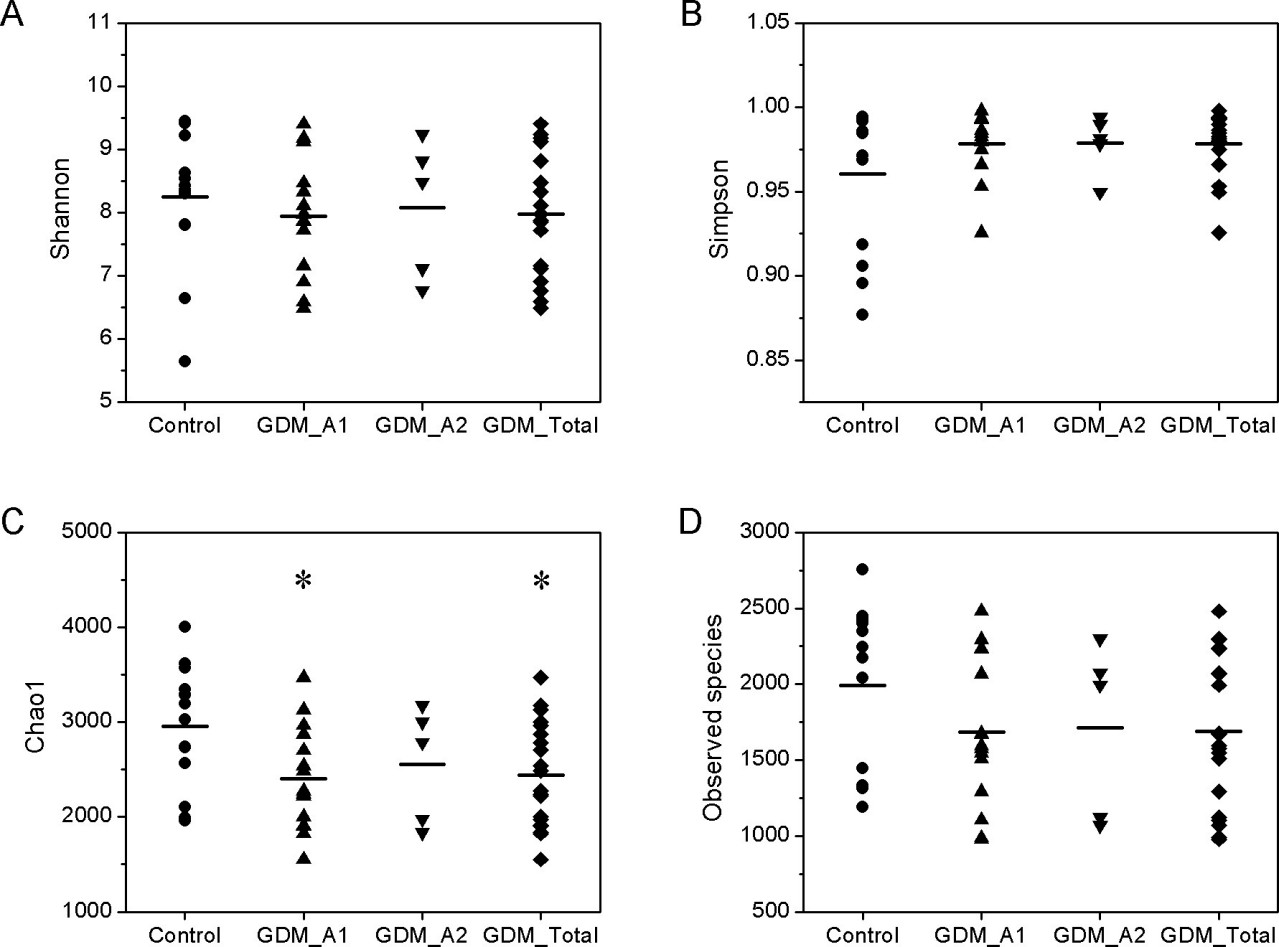
Altered biodiversity of gut microbiota in the GDM groups in comparison with control group. The groups of control (n=14), GDM_A1 (n=15), GDM_A2 (n=5) and GDM_Total (n=A1+A2=20) were described in the Methods. (A, B) The ecological diversity of gut microbiota in the control and GDM groups was measured by Shannon index and Simpson index. (C, D) The alpha-diversity, richness of gut microbiota, was determined by Chao1 index and Observed species index.

The parameters of Shannon index, Simpson index, Chao1 index and Observed species index were used to assess the overall diversity thoroughly. The alpha-diversity, which is the mean species diversity in gut, showed that the gut microbiota from the GDM groups were different to some extent compared to the control group. The Simpson index was used to measure the degree of concentration when individuals were classified into types, and the Shannon index was used to quantify the uncertainty [28]. However, there were no significant differences among the groups in terms of the Shannon and Simpson indexes (Fig 1A and 1B, Wilcoxon test, *P*>0.05). The Chao1 index in the GDM_A1 and GDM_Total group was statistically lower than that in the control group (Fig 1C, Wilcoxon test, *P*<0.05), but the index for the GDM_A2 group was moderately (*P*>0.05) lower than that in the control group. For total number of species, the Observed species index of the control group was moderately higher than that GDM group (Fig 1D, Wilcoxon test, *P*>0.05). These results showed that the number of gut bacteria in the control group was higher than that in GDM group, and there were a large number of dominant bacteria and lower distribution uniformity of bacteria in the GDM group compared to the control group. The number of intestinal bacteria in the GDM group decreased compared with the control group, while the distribution uniformity increased, indicating that some dominant populations were decreasing, especially in GDM grade A1.

**Fig 2:**
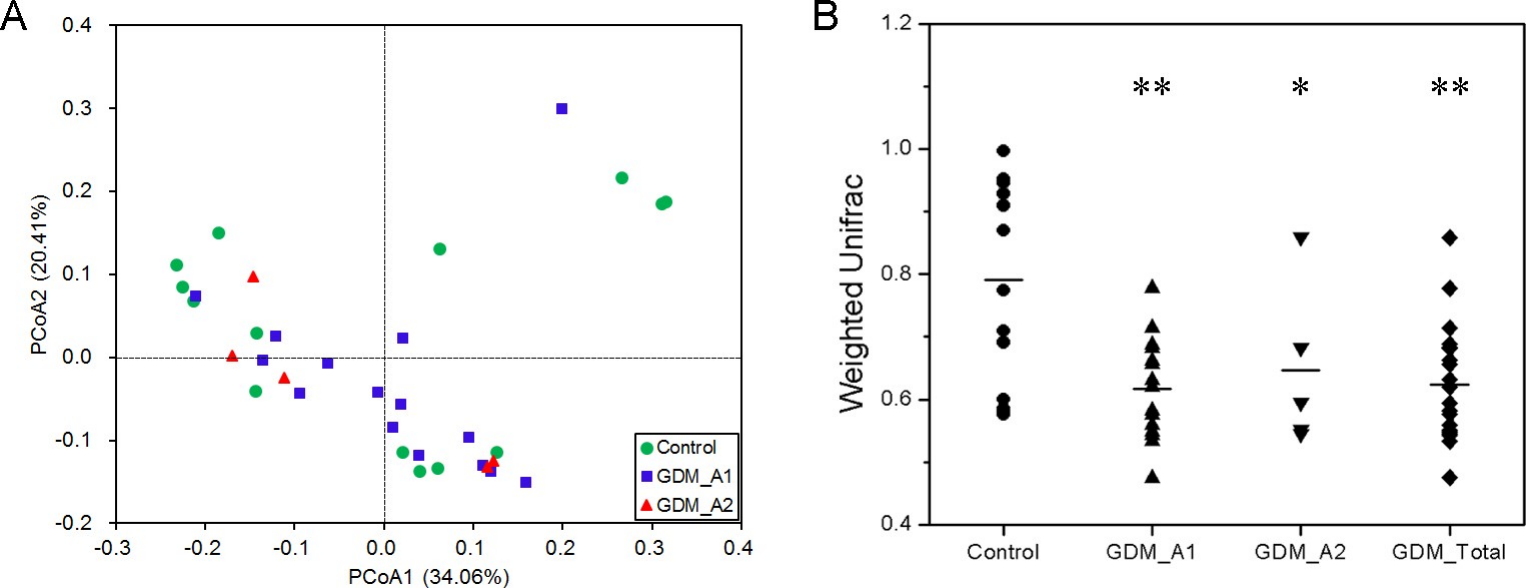
The beta-diversity of the microbial communities in the control and GDM groups. (A) Principal Coordinates Analysis (PCoA) plot based on weighted UniFrac distance. Each dot represents one sample from each group. (B) Beta-diversity index difference based on weighted UniFrac. The *P*-values were calculated using Wilcoxon test. Statistical significance is displayed as * *P* < 0.05 and ** *P* < 0.01.

The results of Weighted UniFrac diversity analysis and PCoA are shown in Fig 2, and indicate that the gut microbial communities were different between the control and GDM groups. The distribution of the control group was more scattered, and the distance between samples was longer than that in GDM group, indicating that individual differences were larger in the control group, and evolutionary similarities of gut microbiota were small and the differences were larger compared with the GDM group (Fig 2A). The two GDM groups had significantly greater PCA variation than that in the control group (Fig 2B, Wilcoxon test, *P*<0.05, *P*<0.01).

### The bacteria differ significantly between the control and GDM groups

**Fig 3:**
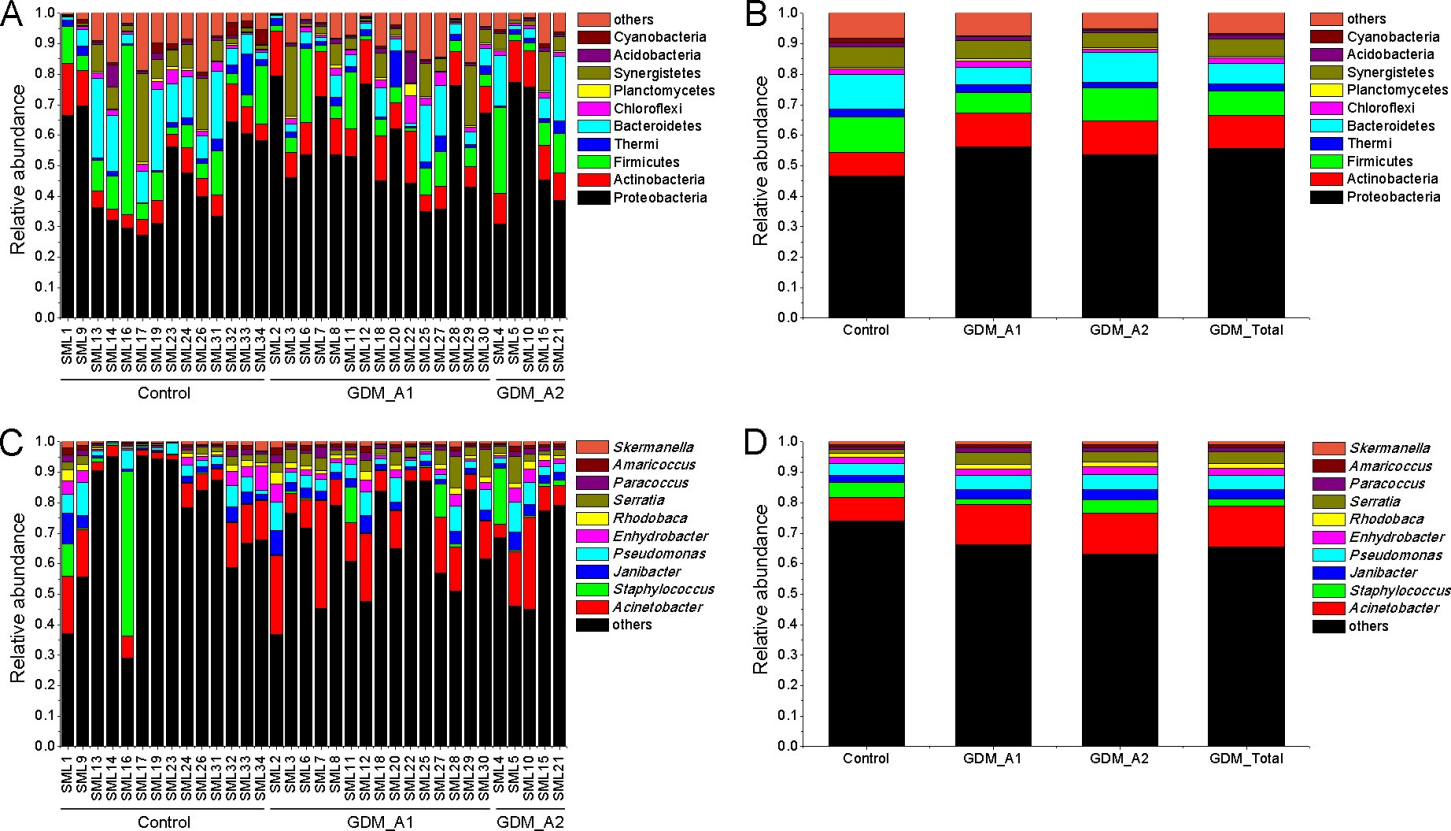
The major gut bacterial phyla and genus in the control and GDM groups. (A) Bacterial phylum levels of each sample in the groups of control, GDM_A1 and GDM_A2 respectively. (B) Bacterial phylum average levels of each group. (C) Bacterial genus levels of each sample in the groups of control, GDM_A1 and GDM_A2 respectively. (D) Bacterial genus average levels of each group.

**Fig 4:**
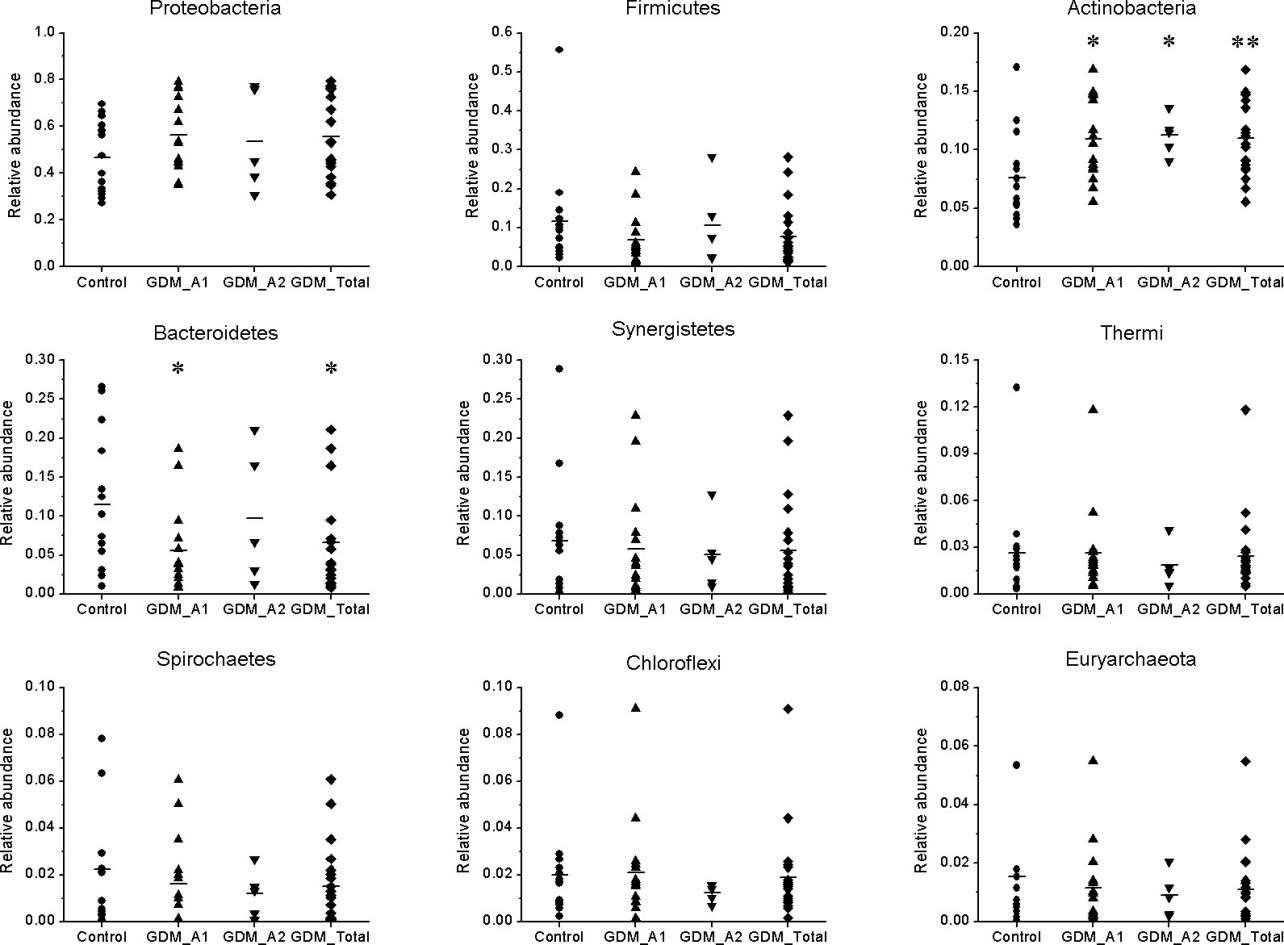
Comparison of gut microbiota of relative abundance at the bacterial phylum levels between GDM groups and control group. The *P*-values were calculated using Mann-Whitney test, and significance was compared against the control group. *P < 0.05. **P < 0.01.

We calculated the number and relative abundance of all species at phylum and genus level among different samples (Fig 3). According to the phylum level, the control and GDM groups both had three phyla of Archaea, and there were 50 and 39 phyla of Bacteria in the control and GDM groups, respectively, showing that more than 90% of species belonged to Bacteria. Bacteria belonging to the phyla of Proteobacteria, Firmicutes, Actinobacteria, Bacteroidetes, Synergistetes, Thermi, Spirochaetes, Chloroflexi and Euryarchaeota were the main gut microbiota in all samples (Fig 4), in which the phyla Proteobacteria was the most prominent of gut microbiota with more than 45% of the total abundance and containing many Gram-negative bacteria and opportunistic pathogenic species. Compared with the control group, relative abundance of Proteobacteria in GDM groups moderately increased, while the Actinobacteria in GDM groups were significantly increased (*P*<0.05, *P*<0.01). Importantly, the phylum Bacteroidetes, which contains many beneficial organisms, was significantly decreased in the GDM_A1 group in comparison with the control group, while the GDM_A2 group had almost no variation. Additionally, the relative abundance of Firmicutes, Synergistetes, Thermi, Spirochaetes, Chloroflexi and Euryarchaeota from the GDM group seemingly decreased compared with that from the control group, however, the decrease did not show any statistical difference between the two groups (*P*>0.05).

Additionally, to understand the difference of gut microbiota at bacterial taxonomic levels of class, order and family, we calculated the amount of gut microbiota between GDM and control group in the main phyla (Figure B in S1 File). Compared with the control group, the amount of gut microbiota at level of class in GDM group significantly decreased in the phyla of Acidobacteria, Chloroflexi and Planctomycetes (*P*<0.05), and the amount of gut microbiota at level of order and family in GDM group significantly decreased in the phyla of Proteobacteria, Firmicutes, Acidobacteria, Chloroflexi and Planctomycetes (*P*<0.05).

**Fig 5:**
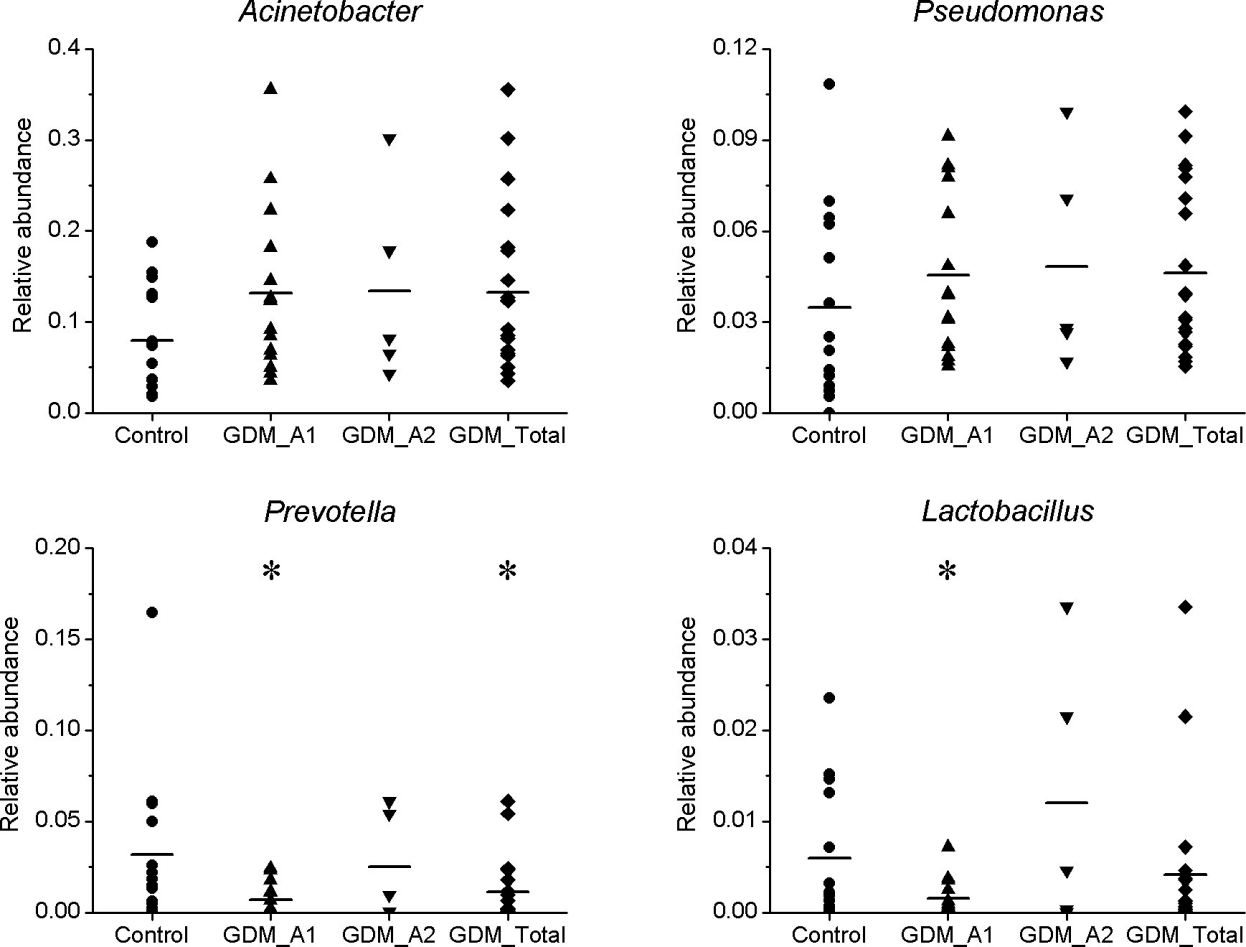
Comparison of gut microbiota of relative abundance at the bacterial genus levels between GDM groups and control group. The *P*-values were calculated using Mann-Whitney test, and significance was compared against the control group. *P < 0.05.

The higher relative abundance of bacterial genera were mainly *Acinetobacter*, *Pseudomonas*, *Prevotella* and *Lactobacillus* in all groups (Fig 5). In general, compared to the control group, genus *Acinetobacter* and *Pseudomonas*, which both belong to the phylum Proteobacteria, and generally contain many opportunistic pathogenic species, moderately increased in the GDM groups. The genus *Prevotella*, which is affiliated with the phylum Bacteroidetes, was significantly decreased (*P*<0.05) in the GDM_A1 group compared to the control group, while the GDM_A2 group had almost no variation. The relative abundance of *Lactobacillus* within the phylum Firmicutes, which is known as a group of major probiotics, was significantly decreased (*P*<0.05) in the GDM_A1 group compared to the control group. The relative abundance of *Lactobacillus* in the GDM_A2 group seemingly increased, however, the increase did not show any statistical difference between the two groups (*P*>0.05).

### The difference of unique gut microbiota between the control and GDM groups

**Fig 6:**
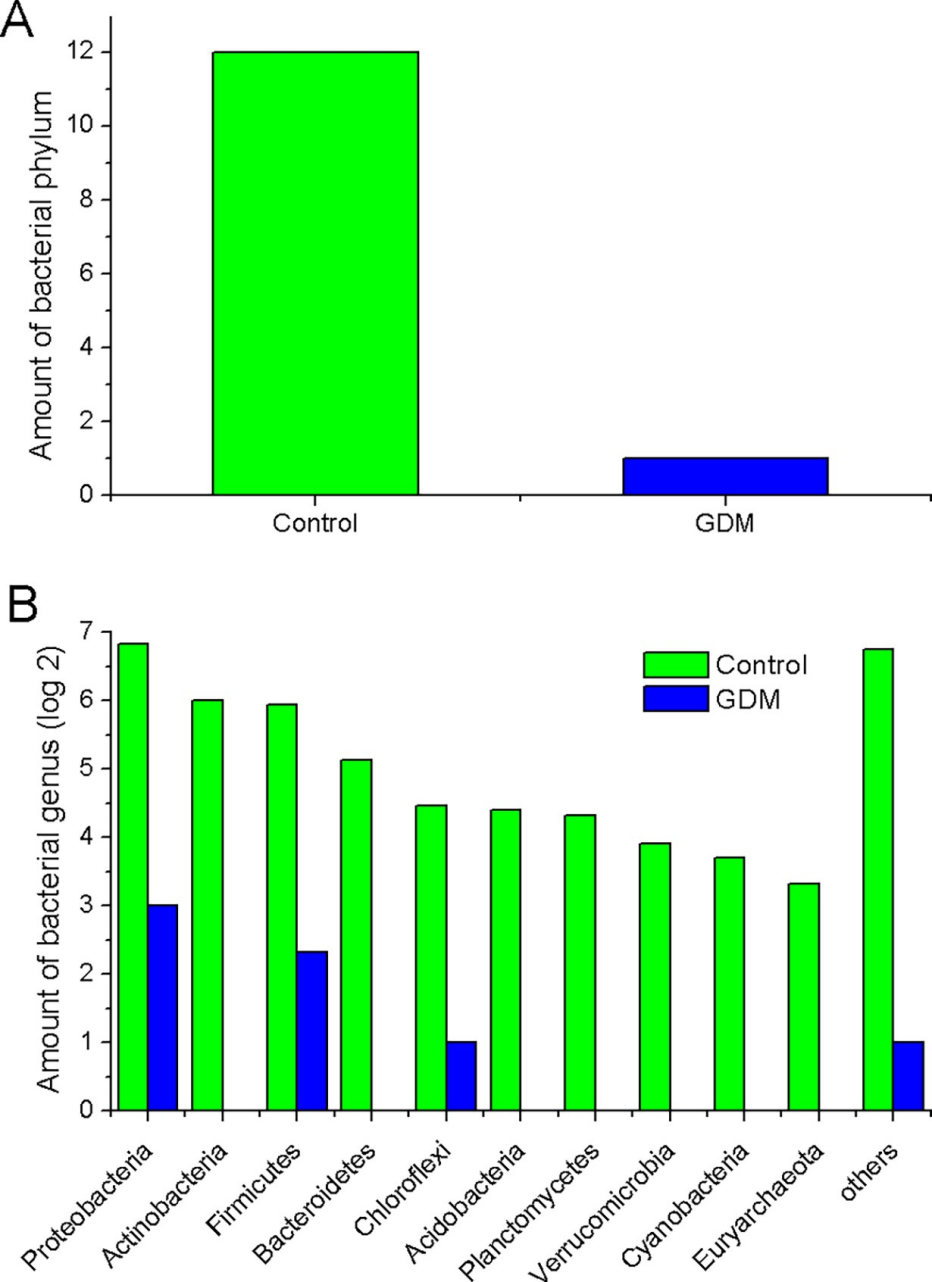
Comparison of the amount of unique gut microbiota at different bacterial taxonomic levels between GDM and control group in the main phyla. (A) Phylum level. (B) Genus level.

At each taxonomic level, the amount of unique gut microbiota between the control and GDM groups are shown in Fig 6. As was shown in Fig 6A, there were 12 unique bacterial phyla in the control group and 1 unique bacterial phylum in the GDM group, with 38 bacterial phyla common in both groups. According to Fig 6B, at the genus level, a large number of bacterial species in the control group could not be found in the GDM group, and conversely, some unique bacterial species in GDM group couldn’t be found in the control group. In the control group, the number of unique bacterial genera in phyla of Proteobacteria, Firmicutes, Actinobacteria, Bacteroidetes, Chloroflexi, Acidobacteria and Planctomycetes were 114, 61, 64, 35, 22, 21 and 20, respectively, which couldn’t be found in GDM group. And in GDM group, the number of the unique bacterial genera in phyla of Proteobacteria, Firmicutes, and Chloroflexi was 8, 5, 2 and 1, respectively, which couldn’t be found in the control group. These unique species may have an association with host metabolism and disease and should be caught on attention. For example, the genus *Butyrivibrio* has been regarded as a key bacterium in polysaccharide degrading and butyrate producing [29], however, it was not detected in GDM group.

### Correlations between maternal fasting glucose levels and gut microbiota in meconium samples of newborns

**Fig 7:**
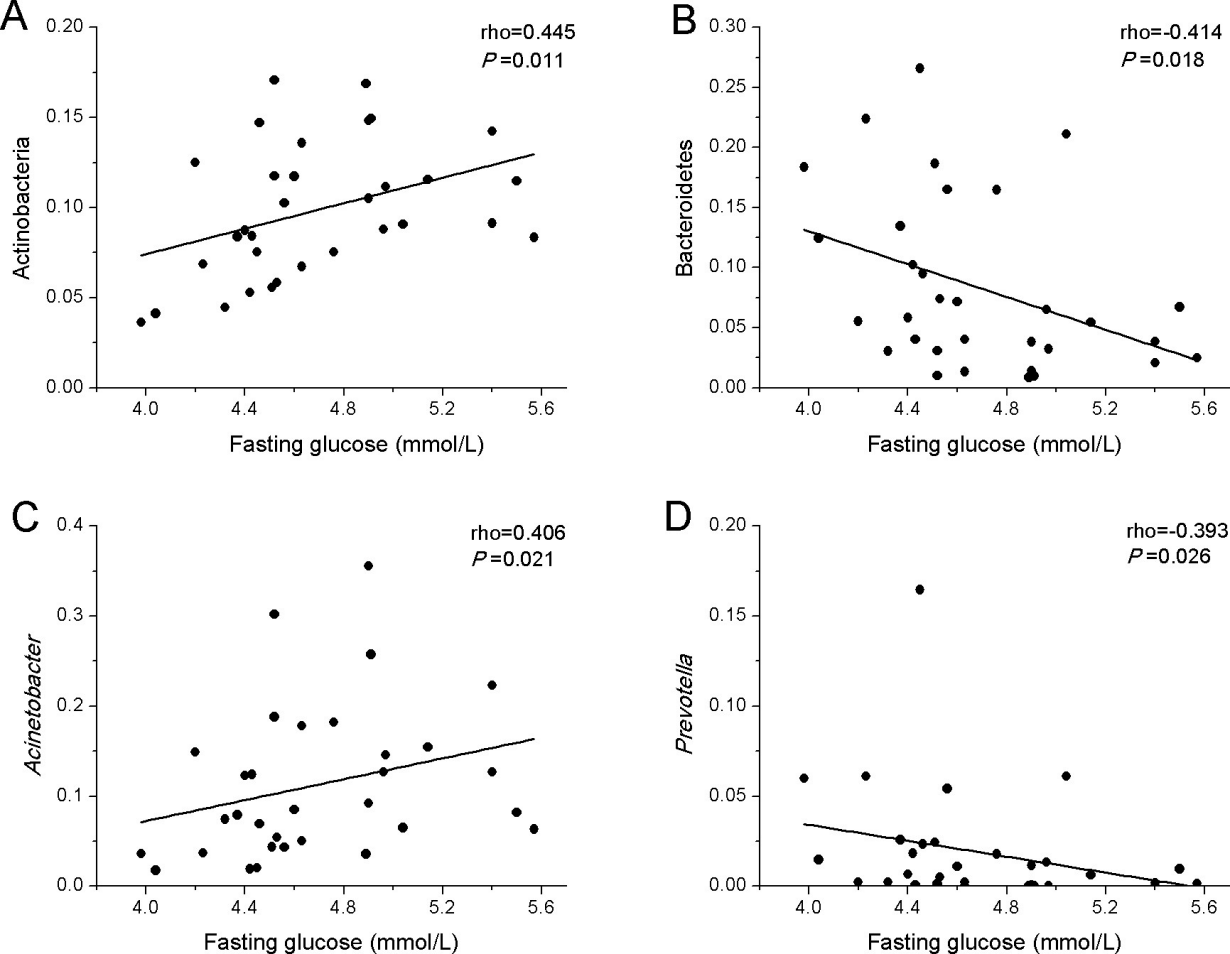
Correlations between maternal fasting glucose and gut microbiota in meconium samples of newborns. Spearman’s rank correlation coefficients and *P*-values for the correlations are shown. (A) Positive correlation between maternal fasting glucose and phylum Actinobacteria (ρ=0.445, *P*=0.011). (B) Negative correlation between maternal fasting glucose and phylum Bacteroidetes (ρ=-0.414, *P*=0.018). (C) Positive correlation between maternal fasting glucose and genus *Acinetobacter* (ρ=0.406, *P*=0.021). (D) Negative correlation between maternal fasting glucose and genus *Prevotella* (ρ=-0.393, *P*=0.026).

To explore whether the maternal glucose metabolism can affect gut microbiota of newborns, correlations between maternal fasting glucose levels and meconium bacteria abundance of newborns were investigated (Fig 7). Results showed that maternal fasting glucose levels were positively correlated with the relative abundance of phylum Actinobacteria (Fig 7A) and genus *Acinetobacter* (Fig 7C), while maternal fasting glucose were negatively correlated with phylum Bacteroidetes (Fig 7B) and genus *Prevotella* (Fig 7D). In addition, we also investigated the correlations between maternal clinical index and meconium bacteria abundance of newborns (Figure C in S1 File). There was negative correlation between maternal age and relative abundance of genus *Lactobacillus* (Figure C in S1A File), while maternal antepartum weight, pre-pregnancy BMI and antepartum BMI were positively correlated with the relative abundance of genus *Clostridium* (Figure C in S1B, S1C and S1D File).

## Discussion

In this study, we successfully sequenced 16S rRNA of meconium samples of newborns from mothers with and without GDM, using the high-throughput sequencing platform. Based on the results of species diversity and taxonomy, we confirm that neonatal meconium was rich in bacteria, regardless of whether the mother was suffering from GDM. Moreover, we demonstrated that gut microbiota from GDM newborns showed a great difference to those from control newborns, with the overall microbiome diversity being significantly lower than that of control newborns (Figs 1 and 2). While at the phylum, class, order, family, and genus level, the amount or relative abundance of gut microbiota from GDM newborns also substantially differed from those from control newborns. In addition, our results showed a positive correlation of maternal fasting glucose with the phylum Actinobacteria and genus *Acinetobacter*, and a negative correlation of maternal fasting glucose with the phylum Bacteroidetes and genus *Prevotella*.

Gut dysbacteriosis not only induces a variety of gastrointestinal diseases, like diarrhoea, constipation and chronic enteritis, but also contributes to the onset and development of many metabolic syndromes and neurogenic diseases, including obesity [30], type 2 diabetes mellitus [31], hypertension [32] and autism [33]. In the present study, we observed a significant decrease in the diversity of various bacterial types of GDM newborns compared to those of normal newborns. These results are consistent with previous findings suggesting that the gut microbiota in the GDM group was associated with lower alpha-diversity compared to healthy groups [34]. The results from the Weighted UniFrac diversity analysis and PCoA (Fig 2) are also consistent with the decreased alpha-diversity, shown in Fig 1. Regarding the species taxonomy, at the phylum level, bacteria belonging to the phyla of Proteobacteria, Firmicutes, Actinobacteria, Bacteroidetes were the first four major gut microbiota in all samples, with the phylum of Proteobacteria being the most abundant of gut microbiota with more than 45% of the total abundance (Fig 4). These results are consistent with the previous findings that the phyla of Actinobacteria, Bacteroidetes, Firmicutes and Proteobacteria were accounted for about 99% of the microbial content in all 23 meconium samples analysed [34]. Furthermore, we observed the phyla of Proteobacteria and Actinobacteria in GDM newborns increased, while the phyla of Firmicutes, Bacteroidetes, Synergistetes, Thermi, Spirochaetes, Chloroflexi, and Euryarchaeota were reduced compared with control infants (Fig 4). It has been well documented that the phylum of Proteobacteria increased in diabetes and obesity, while the phylum of Bacteroidetes decreased [35, 36]. In addition, the amount of a number of bacteria at the level of class, order and family in the GDM group decreased significantly (Figure B in S1 File). Moreover, a large number of unique gut microbiota in the phyla of Proteobacteria, Firmicutes, Actinobacteria, Bacteroidetes, Chloroflexi, Acidobacteria and Planctomycetes from control newborns were absent in GDM newborns. These results indicate that there might be serious dysbacteriosis in the gut of GDM newborns, and they could potentially more prone to suffer from gastrointestinal diseases and metabolic syndrome at subsequent stages in their lives. Clausen et al. have also shown that adult offspring of women with diet-treated GDM or type 1 diabetes are at risk for being overweight and developing metabolic syndromes [37].

Several studies suggest that hyperglycaemia caused by GDM could affect the metabolism of the intrauterine foetus, thus greatly increasing the risk of metabolic syndrome development [38, 39]. Progression of glucose intolerance is accompanied by changes in the proportion and diversity of the gut microbiota [40]. Recently, it was reported that type 2 diabetes in humans was associated with a reduced abundance of butyrate-producing bacteria [31], the decreased levels of which may contribute to diseases [41]. Murri et al. reported a significant decrease in the number of *Lactobacillus* and *Prevotella* in children with diabetes [42]. In the present study, we also observed the relative abundance of *Prevotella* and *Lactobacillus* significantly decreased in GDM newborns (Fig 6). Correlation analysis indicated that maternal fasting glucose levels were negatively correlated with the relative abundance of phylum Bacteroidetes (Fig 7B) and genus *Prevotella* (Fig 7D). *Lactobacillus* has members with probiotic characteristics, which have been associated with positive effects for the host in the large intestine [43]. In addition, many beneficial organic acid lactates produced by *Lactobacillus* could be converted into butyrate by butyrate-producing bacteria in the gut [44]. Previous studies have highlighted the physiological functions of *Prevotella* in maintaining the community structure of human gut microbiome [45, 46]. In addition, another study reported that autism-related gastrointestinal disorders may be linked to the absence of *Prevotella*, especially the *P*. *copri* -like phylotype [47]. Consequently, we speculated that variation of these genera of gut bacteria might be related to diabetes and gastrointestinal disorders, which may pose GDM newborns at a higher risk of developing these diseases in the future than control newborns. It is worth noting that the genera of *Prevotella* and *Lactobacillus* from the GDM_A2 group did not show any statistical variation compared to those from the control group. We speculate that the pregnant women with GDM grade A2 were additionally treated by insulin, which might have affected the gut microbiota of those mothers.

Nevertheless, the impact of GDM treatments and antibiotics contributing to neonatal microbiota need to be further investigated. The maternal treatments might affect infant microbiota, as some researchers have previously described. More molecular sequencing technologies are also recommended for microbe species investigation apart from 16S rRNA sequencing. Likewise, it is suggested to conduct more experiments using RNA sequencing and Metabolomics to investigate how GDM may influence the growth and metabolism of GDM mothers and newborns. In our study, another limitation includes the fact that the sample size may not have allowed us to detect modest differences in bacterial distribution. Therefore, a larger sample size and a functional analysis will also be needed in future studies. Furthermore, may be worth comparing the microbiota of infants and GDM mothers after different treatments of GDM sub-groups.

A previous study has shown that the microbial connection between infant and mother starts before birth, with specific bacteria or bacterial components already observed *in utero* [48]. A rapid emergence of different gut microbiota in the infant occurs shortly after delivery. This stepwise microbiota transfer is expected to be controlled, but it is also affected by external factors, such as weaning, diet habits and environmental exposures to microbes. Several studies have reported that the maternal health status would influence the transfer of microbiota to their offspring [34, 49]. The intestinal microbial community appears to be vulnerable to perturbations during the early life-stages of newborns and it also has fundamental effects on the development of the host immune system [50]. According to our results, the gut microbiota in GDM newborns was significantly different from control newborns. Hence, it is reasonable to assume that regulating the gut microbiota of pregnant women could be a potential path to prevent GDM and decrease the risk of childhood obesity, as well as type 2 diabetes. It has shown that interventions, such as antibiotic treatment or exposure to antibiotics repeatedly, can influence the composition of gut microbiota [51]. Likewise, a certain number of studies have also shown that probiotic supplements are beneficial in the prevention of GDM [52, 53]. However, the evidence for the utilization of antibiotics and probiotics in diagnosed GDM is still scanty and remains to be elucidated.

In conclusion, it has been established that the gut microbiota is an important factor in the onset and development of metabolic diseases [31]. Nevertheless, the specific microbiome components promoting or protecting against these metabolic syndromes and the possible molecular targets of interventions are still under debate. The future of therapeutic potential on the gut microbiota remains to be demonstrated. At present, there is significant ongoing research on microbiome-based therapeutics for gut microbiota worldwide. Fecal microbiota transplant (FMT) has been proven to be effective in treating refractory *Clostridium difficile* infections [54]. Butyrate-producing bacteria preferentially ferment dietary fibers into butyrate and other short chain fatty acids in the colon [55, 56], which are beneficial to the host. It has been reported that butyrate improves insulin resistance and fasting hyperglycaemia by inhibiting adipocyte inflammation [57, 58]. We anticipate that the results of this study may have important implications for understanding the onset, development and optimal treatment of GDM-related diseases by taking effective preventive measures. Investigating the relationship between GDM mother’s and newborn’s gut microbiota could potentially provide more insight on the mechanisms of gut dysbacteriosis.

## Supporting information

S1 File **File includes Figure A, B, and C.** Figure A. Rarefaction curve. Number of observed species identified with increase of sequencing depth in meconium samples of newborns. Figure B. Comparison of the amount of gut microbiota at bacterial taxonomic levels between GDM and control group in the main phyla. (A) Class level. (B) Order level. (C) Family level. The *P*-values were calculated using Mann-Whitney test, and significance was compared against the control group. *P < 0.05. Figure C. Correlations between maternal clinical index and gut microbiota in meconium samples of newborns. Spearman’s rank correlation coefficients and *P*-values for the correlations are shown. (A) Negative correlation between maternal age and genus *Lactobacillus* (ρ=-0.344, *P*=0.047). (B) Positive correlation between maternal antepartum weight and genus *Clostridium* (ρ=0.450, *P*=0.009). (C) Positive correlation between maternal pre-pregnancy BMI and genus *Clostridium* (ρ=0.551, *P*=0.001). (D) Positive correlation between maternal antepartum BMI and genus *Clostridium* (ρ=0.433, *P*=0.012).
(DOC)
