# Supplementary material for: Diversified gut microbiota in newborns of mothers with gestational diabetes mellitus
Source: PLoS One. 2018 Oct 17;13(10):e0205695. doi: 10.1371/journal.pone.0205695 (PMC6192631; doi:10.1371/journal.pone.0205695)
Supplement: S1 File — File includes Figure A, B, and C. Figure A. Rarefaction curve. Number of observed species identified with increase of sequencing depth in meconium samples of newborns. Figure B. Comparison of the amount of gut microbiota at bacterial taxonomic levels between GDM and control group in the main phyla. (A) Class level. (B) Order level. (C) Family level. The P-values were calculated using Mann-Whitney test, and significance was compared against the control group. *P < 0.05. Figure C. Correlations between maternal clinical index and gut microbiota in meconium samples of newborns. Spearman’s rank correlation coefficients and P-values for the correlations are shown. (A) Negative correlation between maternal age and genus Lactobacillus (ρ=-0.344, P=0.047). (B) Positive correlation between maternal antepartum weight and genus Clostridium (ρ=0.450, P=0.009). (C) Positive correlation between maternal pre-pregnancy BMI and genus Clostridium (ρ=0.551, P=0.001). (D) Positive correlation between maternal antepartum BMI and genus Clostridium (ρ=0.433, P=0.012). (DOC) [file pone.0205695.s001.doc]

**Supporting information**

**
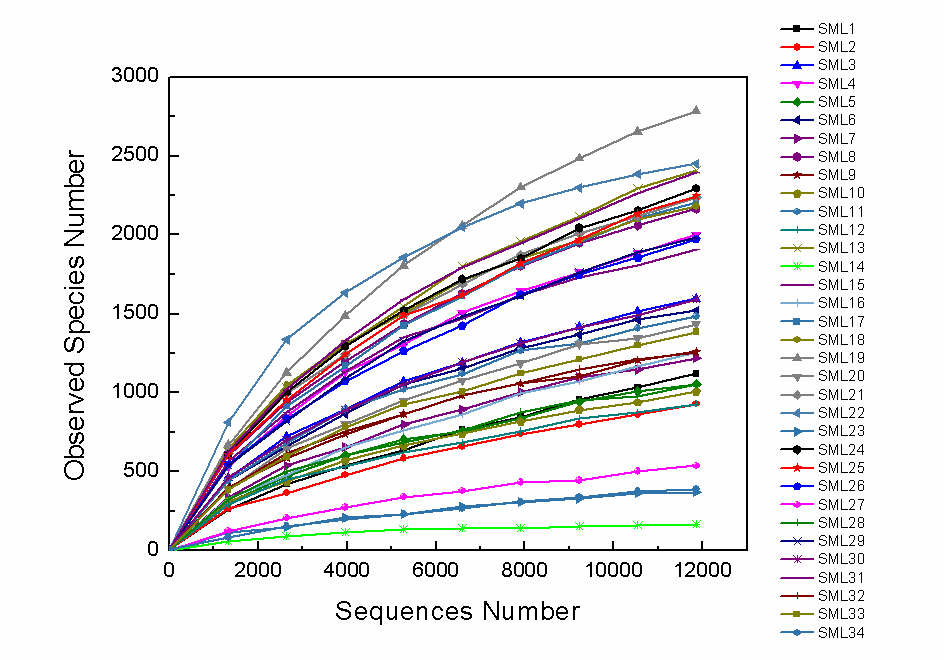
**

**Figure A.** **Rarefaction curve.** Number of observed species identified with increase of sequencing depth in meconium samples of newborns.


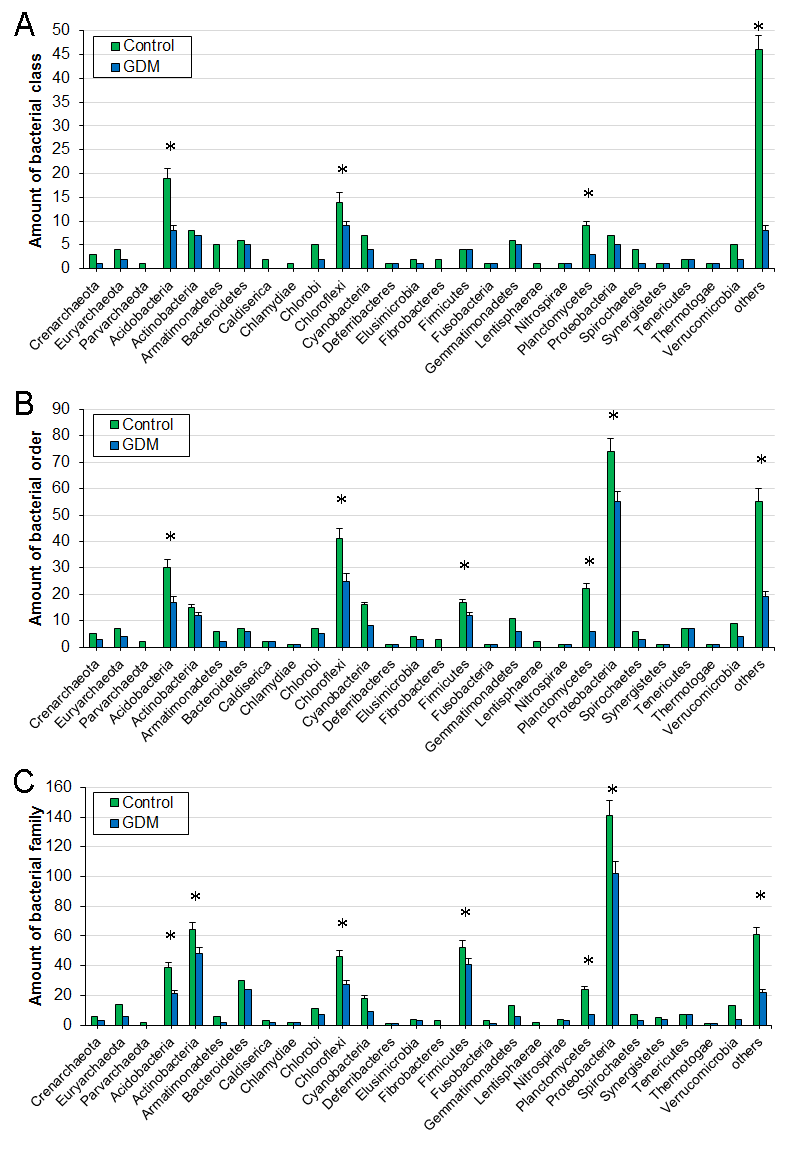


**Figure B.** **Comparison of the amount of gut microbiota at bacterial taxonomic levels between GDM and control group in the main phyla.** (A) Class level. (B) Order level. (C) Family level. The *P*-values were calculated using Mann-Whitney test, and significance was compared against the control group. **P* < 0.05.


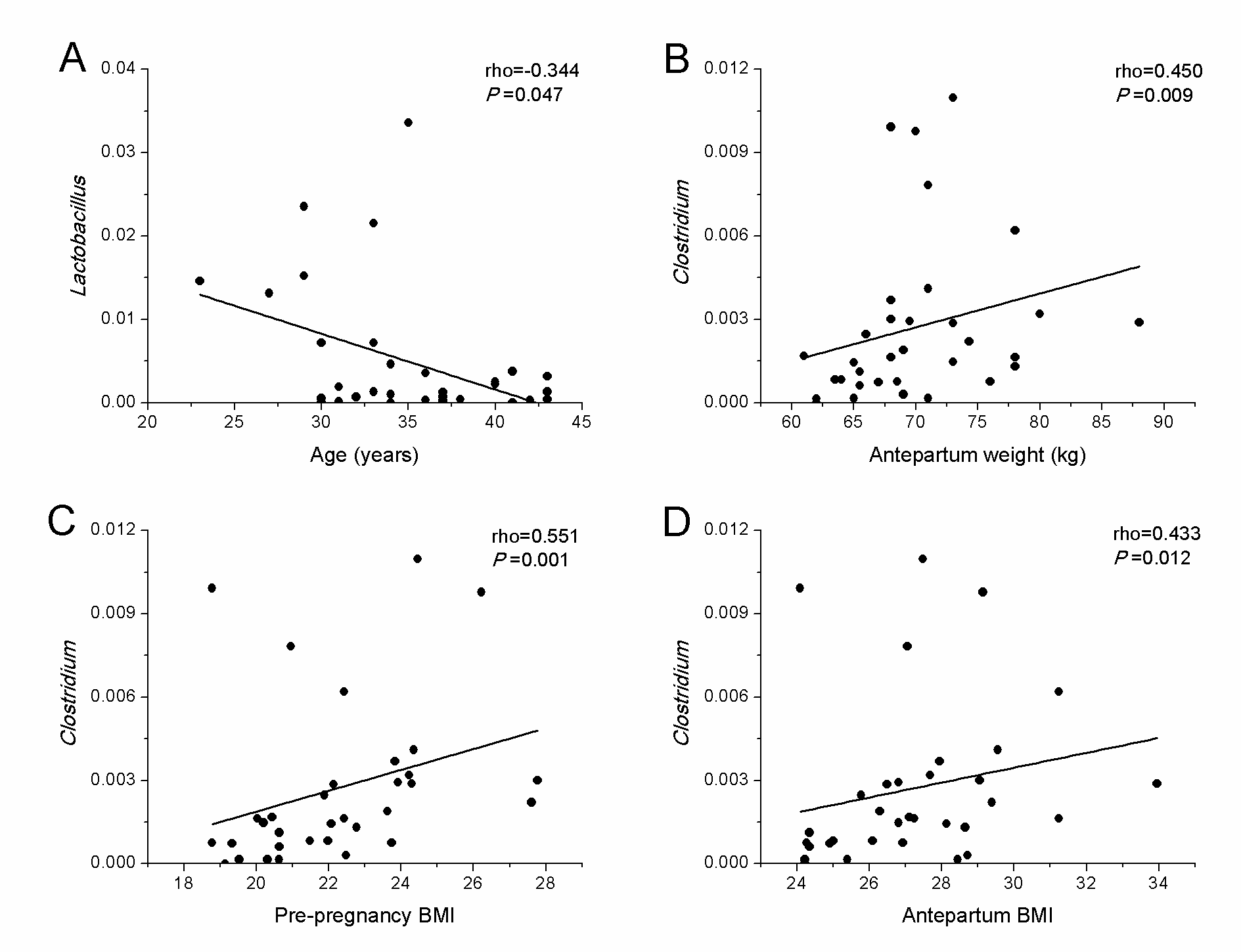


**Figure C.** **Correlations between maternal clinical index and gut microbiota in meconium samples of newborns.** Spearman’s rank correlation coefficients and *P*-values for the correlations are shown. (A) Negative correlation between maternal age and genus *Lactobacillus* (ρ=-0.344, *P*=0.047). (B) Positive correlation between maternal antepartum weight and genus *Clostridium* (ρ=0.450, *P*=0.009). (C) Positive correlation between maternal pre-pregnancy BMI and genus *Clostridium* (ρ=0.551, *P*=0.001). (D) Positive correlation between maternal antepartum BMI and genus *Clostridium* (ρ=0.433, *P*=0.012).
